# Longitudinal Study of Mammary Epithelial and Fibroblast Co-Cultures Using Optical Coherence Tomography Reveals Morphological Hallmarks of Pre-Malignancy

**DOI:** 10.1371/journal.pone.0049148

**Published:** 2012-11-12

**Authors:** Raghav K. Chhetri, Zachary F. Phillips, Melissa A. Troester, Amy L. Oldenburg

**Affiliations:** 1 Department of Physics and Astronomy, University of North Carolina at Chapel Hill, Chapel Hill, North Carolina, United States of America; 2 Curriculum in Applied Sciences and Engineering, University of North Carolina at Chapel Hill, Chapel Hill, North Carolina, United States of America; 3 Lineberger Comprehensive Cancer Center/Department of Epidemiology, University of North Carolina at Chapel Hill, Chapel Hill, North Carolina, United States of America; 4 Biomedical Research Imaging Center, University of North Carolina at Chapel Hill, Chapel Hill, North Carolina, United States of America; Fox Chase Cancer Center, United States of America

## Abstract

The human mammary gland is a complex and heterogeneous organ, where the interactions between mammary epithelial cells (MEC) and stromal fibroblasts are known to regulate normal biology and tumorigenesis. We aimed to longitudinally evaluate morphology and size of organoids in 3D co-cultures of normal (MCF10A) or pre-malignant (MCF10DCIS.com) MEC and hTERT-immortalized fibroblasts from reduction mammoplasty (RMF). This co-culture model, based on an isogenic panel of cell lines, can yield insights to understand breast cancer progression. However, 3D cultures pose challenges for quantitative assessment and imaging, especially when the goal is to measure the same organoid structures over time. Using optical coherence tomography (OCT) as a non-invasive method to longitudinally quantify morphological changes, we found that OCT provides excellent visualization of MEC-fibroblast co-cultures as they form ductal acini and remodel over time. Different concentrations of fibroblasts and MEC reflecting reported physiological ratios [1] were evaluated, and we found that larger, hollower, and more aspherical acini were formed only by pre-malignant MEC (MCF10DCIS.com) in the presence of fibroblasts, whereas in comparable conditions, normal MEC (MCF10A) acini remained smaller and less aspherical. The ratio of fibroblast to MEC was also influential in determining organoid phenotypes, with higher concentrations of fibroblasts producing more aspherical structures in MCF10DCIS.com. These findings suggest that stromal-epithelial interactions between fibroblasts and MEC can be modeled *in vitro*, with OCT imaging as a convenient means of assaying time dependent changes, with the potential for yielding important biological insights about the differences between benign and pre-malignant cells.

## Introduction

The human mammary gland consists of a series of branching ducts, with each branch terminating as a hollow and spherical acinus. Each acinus is predominantly comprised of luminal epithelial cells surrounded by basal/myoepithelial cells, but is supported and regulated by an intricate network of other cell types. Chemical and physical interactions between epithelia and surrounding stroma are essential for the organ’s development and physiological functions. This intricate network of cells is a complex microenvironment [2] that maintains normal tissue architecture (homeostasis) and suppresses malignant phenotypes in healthy individuals [3], but becomes permissive or even promotes cancer during progression [4]. Thus, interactions between mammary epithelial cells (MEC) and stromal fibroblasts are regulators of tumorigenesis [4], [5], with stroma playing a vital role in the proliferation and organization of MEC, production of extracellular matrix (ECM), and regulation of cellular adhesion and migration [4].

Fibroblasts are strongly associated with mammary epithelium, and in the vicinity of tumors, become a major cell type of the stroma [6]. These cancer-associated fibroblasts appear to promote tumor growth and facilitate the progression of breast cancer [7]. Conversely, normal fibroblasts may inhibit progression of cancer [8]. Our previous studies have illustrated that fibroblasts have distinct interactions with breast cancer subtype [9], with aggressive basal-like breast cancer cells [10] interacting with fibroblasts to produce a wide range of growth factors and cytokines that may in turn promote migration and/or proliferation of the cancer cells. However, the evolution of these interactions during breast cancer progression has not yet been well characterized. By comparing normal and pre-malignant MEC co-cultured with RMF in 3D, and by modulating the ratios of the two cell types, we aimed to elucidate how stromal-epithelial interactions modulate morphological changes in acini.

Our previous studies on interactions between breast cancer cells and fibroblasts have relied on 2D cultures [9], but 3D co-culture models offer an interface between these studies and *in vivo* studies given their ability to recapitulate several aspects of tissue behavior [11]–[13]. Novel tools that image the 3D breast microenvironment can elucidate micron-scale morphological changes during the dynamic chemical and physical signaling processes between mammary cell types. To date, a majority of the studies of stromal-epithelial co-cultures have utilized imaging techniques that require sample fixation and often sectioning [14], which can perturb the native architecture and present challenges for longitudinal studies. To address these limitations, optical coherence tomography (OCT), which can assess cellular dynamics in 3D tissue models [15], was employed to non-invasively capture the 3D architecture of breast tissue models.

OCT represents an emerging medical and biological optical imaging modality [16]–[19], that performs cross-sectional imaging of internal microstructures in tissues by measuring the magnitude and echo time delay of backscattered, near-infrared light. OCT provides micron-scale resolution for cellular imaging, and rejects multiply scattered light, unlike confocal microscopy, which enables imaging up to 2–3 millimeters in depth. This depth is ideal for assessing subsurface structures such as 3D tissue cultures [15]. Simultaneously, the non-invasive nature of OCT enables longitudinal studies in the same samples, avoiding the need to excise and process tissue specimens [20]. Recent studies have demonstrated the feasibility of OCT to provide image-guidance by scanning tumor margins during breast-sparing surgery [21], [22], and to identify invasive breast carcinomas in biopsy tissue [23]. OCT imaging has also been employed on unstained, *ex vivo* breast cancer tissues to identify morphological features, similar to histology [24], [25]. Additionally, computational methods to perform pattern analysis of OCT biopsies have been implemented to identify invasive breast carcinomas [26], [27]. Thus, OCT has translational potential with applications in basic studies and *in vivo* clinical imaging. As such, OCT imaging offers a unique platform for evaluating the architecture of MEC grown in 3D co-cultures.

The aim of this study was to define morphological hallmarks of stromal-epithelial interactions using OCT to assess 3D *in vitro* cultures comprised of basal-like mammary epithelial cell lines (normal MCF10A, and pre-malignant MCF10DCIS.com) [28] and hTERT-immortalized fibroblasts from reduction mammoplasty (RMF). As shown below, we found distinct morphological features between acini formed by normal MCF10A cells and pre-malignant MCF10DCIS.com cells as a function of fibroblast concentration.

## Methods

### Cell Lines

MCF10A and MCF10DCIS.com cells were obtained from the Barbara Ann Karamanos Cancer Institute (Detroit, MI). MCF10A cells are spontaneously immortalized MEC derived from the human breast tissue of a 36-year-old patient [29], and exhibit numerous features of normal breast epithelium including lack of tumorigenicity and dependence on growth factors and hormones for proliferation and survival [29]. Importantly, MCF10A cells in 3D cultures form stable acinar structures recapitulating the behavior of glandular epithelium seen *in vivo* [30]. MCF10DCIS. com cells are cloned from xenograft lesions of MCF10A and form DCIS-like lesions [31]. Importantly, MCF10DCIS.com cells have the same genetic background as the MCF10A, and are primed for invasive transition under key microenvironmental conditions, requiring no additional genomic changes to become invasive [31]. The MCF10A and MCF10DCIS.com cells were co-cultured with hTERT-immortalized fibroblasts from reduction mammoplasty (RMF), a gift from Charlotte Kuperwasser at Tufts University [32]. All cells used in this experiment were maintained prior to use in 2D cultures in Dulbecco's Modified Eagle Medium/Nutrient Mixture F-12 (DMEM/F12) containing 5% horse serum, 20 ng/mL Epidermal Growth Factor (EGF), 0.5 µg/mL hydrocortisone, 100 ng/mL cholera toxin, 10 µg/µL insulin, and 1% penicillin-streptomycin, and kept in a humidified incubator at 37°C and 5% CO_2_ [12].

### 3D Culture Preparation

The 3D extracellular scaffold used in this study consisted of biologically derived collagen I and Matrigel® (BD Biosciences). Compared to collagen I gels, Matrigel-collagen I gels were found to be structurally more stable and thus less prone to loss over the duration of the study due to several cycles of media replenishments, as has been previously noted [33]. For 3D cell culture, a Matrigel-collagen I mixture was prepared on ice using a 1∶1volume ratio, with collagen I at a concentration of 1 mg/mL, according to procedures described by Johnson *et. al*. [34]. Once the MEC and RMF were nearly 100% confluent in 2D, they were seeded at varying concentrations into the Matrigel-collagen I gel for growth in 3D, as follows: A total of 27 3D cultures were prepared, which included 9 co-cultures of normal MEC and RMF, 9 co-cultures of pre-malignant MEC and RMF, and 3 monocultures each of normal MEC, pre-malignant MEC, and RMF. Briefly, the following protocol was used for all co-cultures. 85 µL of Matrigel-collagen I was used to coat the bottom of 10 mm diameter tissue culture microwells, and was allowed to solidify for 30 minutes at 37°C. Then, 180 µL of Martrigel-collagen I gel was mixed with MEC and RMF according to procedures described in [9] to obtain the desired final seed concentrations. The seed concentrations of MEC and RMF in the Martigel-collagen I gels were varied as 30,000 cells/cm^3^, 90,000 cells/cm^3^, 270,000 cells/cm^3^ and control, and were plated and allowed to solidify for 30 minutes at 37°C. After gelation, 250 µL of growth media (same as in 2D cultures above) was applied to the surface of each 3D culture. Cultures were maintained under optimum growth conditions (humidified, 37°C with 5% CO_2_) for 2–4 weeks, during which the medium was changed every 2–3 days.

Although the co-cultures were maintained for 4 weeks (Figure S4, Table S1), proliferation of the cells in the co-cultures was no longer in the log-phase of growth after week 2, as evidenced by a plateau in the number of MEC per acinus (Figure S5). Similarly, co-cultures with higher seeded cell concentration (MEC concentration >90,000/cm^3^) also remained in log-phase for only a short time (Figure S5). We selected only monocultures and co-cultures that were still in log-phase to avoid artifacts in morphology caused by resource scarcity or cellular crowding.

### OCT Imaging

Imaging of the 3D cultures was performed using a custom, ultrahigh-resolution, spectral-domain optical coherence tomography (SD-OCT) system as described in detail previously [35]. The OCT system employed a low-coherence light source consisting of a Ti:Sapphire laser (Griffin, KMLabs, Inc.) with a central wavelength of 800 nm and a bandwidth of 125 nm. A detailed description of the OCT system and the system diagram is provided in the supplementary (Figure S1). The axial (depth, *z*) resolution of the imaging system owing to the wavelength and the bandwidth of the light source is 3 µm in air. In the sample arm, 3D cultures were illuminated by a 10 mW beam focused by a 30 mm focal length achromatic lens, which provides a resolution of 12 µm (air) in the transverse (*x* and *y*) directions. Transverse raster-scanning over the sample was achieved using galvanometer-controlled mirrors. OCT imaging was performed on each of the live 3D cultures weekly for 4 weeks. OCT image-stacks were acquired over 3×1.5×1.5 mm (in gel) into 1000×101×1024 pixels (*x*, *y*, and *z* dimensions respectively) with an acquisition time of 40 ms per *x-z* image. The OCT image-stacks were resampled into an isotropic pixel resolution of 1.55 µm after correcting for the refractive index of the aqueous gels, and are logarithmically scaled and displayed in a “hot” color map using MATLAB® (2011a, MathWorks).

### Image Analysis

**Figure 1:**
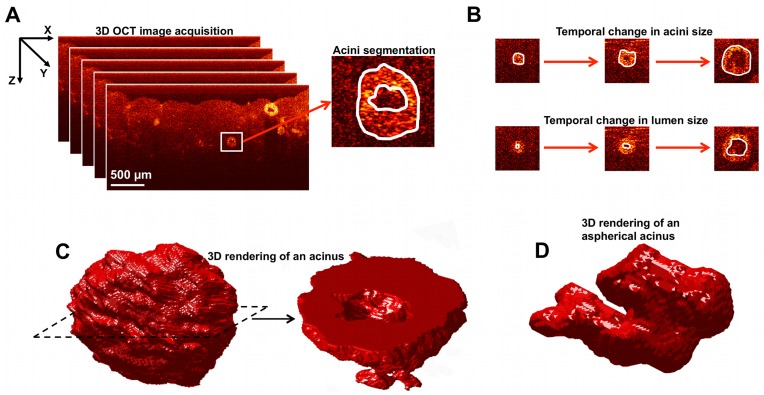
3D-OCT image acquisition of the co-cultures, and analysis of the shape and size of acini. A. 3D-OCT image acquistion: the surface of the gel is aligned near the top of each image, and the depth-resolved light scattering from cells beneath the gel surface is apparent at depths up to ∼1 mm; segmentation of acini to characterize the overall size and the lumen is also shown. B. Temporal changes in acini and lumen sizes analyzed from 3D-OCT images of the co-cultures. C. An example isosurface rendering of an acinus from a 3D-OCT image-stack; slicing of the rendered volume clearly shows the lumen. D. An example 3D rendering of an aspherical acinus.

2D analysis of OCT images was performed to determine the maximum acinar and lumen areas. From the color-mapped OCT images, cell clusters resembling acini were selected as shown in Figure 1A. The OCT image containing the central position of each acinus was determined by sifting through the OCT image-stack to find the image with the largest acinus size. The overall acinus area (cells plus lumen) and lumen area were segmented within these central OCT images using ImageJ, as shown in Figure 1B. The results were tabulated for each culture, from which the mean acini area, mean lumen area, and their associated standard errors were evaluated.

As depicted in Figures 1C and 1D, iso-surface rendering of the OCT image stacks enables visualization of the entire 3D acinar structure. In order to quantify the 3D morphology of the acini, we computed the asphericity, that is, the deviation in acini shape from that of a perfect sphere. We defined asphericity as the ratio between the volume of a perfect sphere having the same surface area as that of the acinus, *S_acini_*, and the measured volume of each acinus, *V_acini_*, according to [36]:
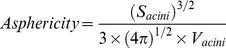
(1)where *S_acini_* is in pixels^2^, and *V_acini_* is in pixels^3^. Asphericity is equal to 1 for a perfect sphere and increases for irregularly shaped objects. Thus, asphericity provides a size-independent measurement of how aspherical a 3D structure is, which aids in quantifying surface irregularities across renderings of various sizes.

To compute *V_acini_*, OCT images were first median filtered, and a 3D mask of each acinus was obtained by thresholding the OCT image stack. Then, the ‘bwboundries’ command in MATLAB was applied to each 2D image in the stack to find the boundaries of thresholded objects and to “fill in” any child objects, such as the lumen. Subsequently, stray objects that did not have any voxel overlap with the acini were removed from the 3D mask, to obtain just the acini. The volume, *V_acini_*, was then computed by counting the voxels comprising the mask. Using simulated data with known geometries, it was verified that this method accurately estimated the volume of the 3D rendered object.

To compute *S_acini_*, the voxels comprising the boundary of the 3D mask were counted. Due to digitization noise, a pixelation correction factor of 1.5 was needed to obtain the correct value of surface area, which corrects for pixel connectivity. This was determined in MATLAB by comparing the measured surface area to the actual surface area of spheres and ellipsoids. Above a radius of 15 pixels and for values of asphericity from 1–8, the pixelation correction factor converged to 1.5; all acini analyzed in this study had radii and asphericities within these valid ranges.

## Results

OCT offers excellent visualization of cellular acini, as shown with representative OCT *x-z* images of the cell cultures in 3D matrices at 1 week (Figure 1A). Representative images in all 3D cultures at weeks 1 and 2 can be seen in supplementary (Figure S2 and S3). At week 1, the MEC organize into spherical clusters (acini) with some clusters showing signs of a lumen at the center (Figure 1B, representative example). At week 2, the acini are observed to have grown in size, displaying larger and more distinct central lumens. The control gel with no cells shows a homogeneous Matrigel:collagen I structure, which was stable throughout the duration of the study. As expected, no spherical clusters were observed in RMF monocultures. Instead, RMF monocultures revealed a fibrous optical scattering pattern characteristic of fibroblasts. As the RMF concentration was increased, a higher density of fibrous structures was observed, corresponding to added rigidity of the matrix.

OCT images of co-cultures reveal a complex pattern of growth and interaction between RMF and the MEC, similar to *in vivo* mammary architecture. Importantly, unlike other techniques that involve slicing, fixing and staining of the gels, these images depict the unperturbed states of the live MEC and RMF *in vitro*. Thus the images were used to measure lumen size, acini size (Figure 1B) and to estimate the shape, characterizing cells on a continuum between spherical (*e.g*. in Figure 1C) and aspherical (*e.g*. in Figure 1D).

**Figure 2:**
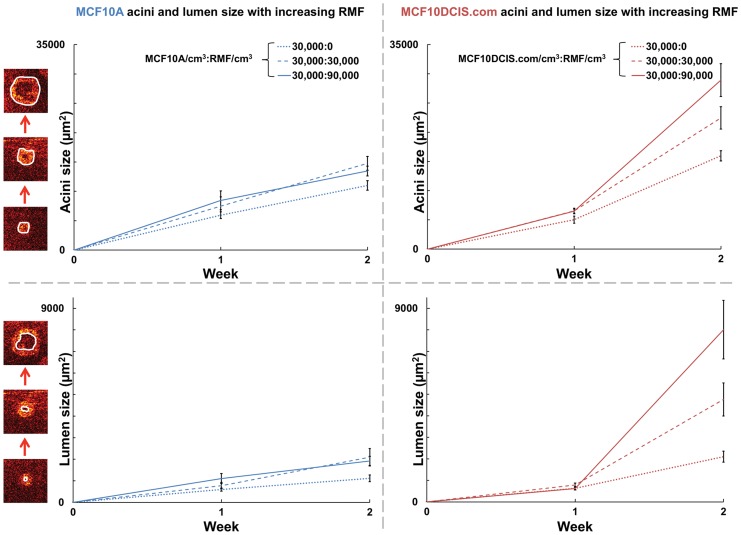
Acini and lumen size. Comparison of MCF10A:RMF co-cultures with MCF10DCIS.com:RMF co-cultures shows significantly larger acini and lumen sizes (Student’s t-test, p-value <0.005) at week 2. In MCF10DCIS.com:RMF co-cultures, acini and lumen size are also observed to be highly modulated by the ratio of fibroblasts.

During the first two weeks of the study, both acini and lumen sizes increased (Figure 2). In normal MEC, the stromal:epithelial ratio did not impact acini and lumen sizes; co-cultures were seeded with 30,000 MCF10A/cm^3^, and as the seed concentration of RMF was increased from 30,000 RMF/cm^3^ to 90,000 RMF/cm^3^, no significant difference was seen in acini sizes (Student’s t-test, p-value = 0.43) or lumen sizes (Student’s t-test, p-value = 0.71) at week 2. However, the size of pre-malignant MEC acini varied in association with stromal content. In co-cultures seeded with 30,000 MCF10DCIS.com/cm^3^, as the seed concentration of RMF was increased from 30,000 RMF/cm^3^ to 90,000 RMF/cm^3^, statistically significant differences were seen in acini size (Student’s t-test, p-value <0.05) and lumen size (Student’s t-test, p-value <0.05) at week 2. In addition, comparing MCF10A to MCF10DCIS acini at week 2, MCF10DCIS.com:RMF co-cultures showed significantly larger acini and lumen sizes across the same seed concentrations (Student’s t-test, p-value <0.005). The stimulatory effect of increased fibroblast concentration on pre-malignant MCF10DCIS.com suggests unique molecular and/or mechanical interactions that stimulate abnormal growth that are not observed in the MCF10A cells.

**Figure 3:**
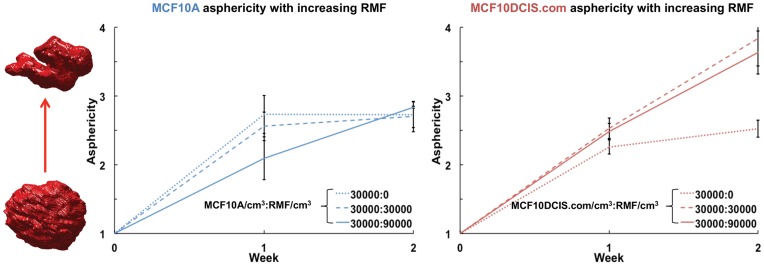
As phericity of acini. The minimum asphericity value of 1 indicates a perfect sphere, while less spherical acini have higher asphericity values. Acini comprised of MCF10DCIS.com cells are seen to become increasingly aspherical in the presence of fibroblasts.

In addition to changes in acini and lumen sizes, MCF10DCIS.com cells also responded to co-culture with increasingly aspherical structures. Since asphericity is a size-independent metric, as expected, no significant correlation was found between asphericity and acini or lumen sizes in both MCF10A and MCF10DCIS.com cultures. Figure 3 shows the asphericity of the rendered acini in monocultures of MCF10A, MCF10DCIS.com, and co-cultures of these cells with increasing concentrations of RMF (30,000 RMF/cm^3^ to 90,000 RMF/cm^3^). Again, in normal cells at week 2, fibroblasts did not affect asphericity; monoculture seeded with 30,000 MCF10A/cm^3^ and co-cultures seeded with 30,000 MCF10A/cm^3^ had similar asphericity values. In contrast, at week 2, the MCF10DCIS.com:RMF co-cultures seeded with 30,000 MCF10DCIS/cm^3^ had significantly increased asphericity relative to monocultures (Student’s t-test, p-value <0.005). Thus, acini formed by pre-malignant MCF10DCIS.com cells in the presence of RMF undergo a higher degree of shape difference than do acini formed by normal MCF10A cells. The observed higher asphericity values in MCF10DCIS.com acini compared to MCF10A acini in presence of RMF highlights the role of fibroblasts in varying the morphology of the acini.

In summary, these observations show that fibroblasts differentially modulate the shape and size of acini comprised of pre-malignant and normal MEC.

## Discussion

The application of OCT to 3D co-cultures of MEC and RMF provided excellent visualization of acinar development over time and recapitulation of *in vivo* morphologies. Acini formed by MCF10A cells in 3D co-cultures were found to be comparable in size to freshly explanted acini previously reported [30]. Observations of increased acini size, lumen size, and asphericity in pre-malignant MCF10DCIS.com co-cultures compared to normal MCF10A co-cultures, and the distinct influence of fibroblast concentration on these phenotypes, suggest that changes over time in stromal-epithelial interactions in 3D co-culture models can be detected using OCT. Interestingly, while acini morphogenesis in DCIS *in vivo* is typically envisioned as progressive invasion into the lumen by the proliferating carcinoma cells [6], our non-invasive study of live 3D co-cultures revealed no such invasion of MCF10DCIS.com into the luminal spaces. However, the formation of luminal space was expected based on *in vivo* studies, as the MCF10DCIS.com cells are comedo-type DCIS [37]. This reinforces the advantage of using OCT to non-invasively and longitudinally probe the same live co-cultures; traditional techniques require slicing, fixing and staining of repeat co-cultures at each time point, which can perturb the natural state and architecture of the organoids. A previous 3D tissue study in MEC monocultures has identified four distinct breast cell line colony morphologies: round, mass, grape-like and stellate [13]. In that study, MCF10A acini are identified as round. A comparatively gentle treatment of the 3D cultures may account for the ability of this system to detect morphology reflective of the unique *in vivo* characteristics of comedo-type DCIS. The ability to regularly probe the same samples longitudinally also avoids problems associated with sample-to-sample variability. In addition, OCT enabled rapid collection of volumetric data with a scan time of 90 seconds per culture, which, at the time of the study, was limited by software and has recently been sped to 4 seconds per culture.

Previous research has highlighted differences between mechanical stromal-epithelial interactions (MEC and fibroblasts in physical contact), and chemical stromal-epithelial interactions (MEC and fibroblasts separated by a barrier allowing passage of soluble signals) [9]. Our results further indicated a difference in stromal-epithelial interactions between fibroblasts and normal or pre-malignant MEC, as evidenced by pronounced differences in morphological features. A number of previous studies have demonstrated that co-cultures with DCIS cells can provide interesting insights regarding signaling and phenotypes of malignant progression [38]. Indeed, our co-cultures mirrored many of the phenotypes previously observed in mammary epithelial monocultures [12], [39], while also providing fibroblast-dependent morphological change over time in the same samples. The ability to study phenotypes over time offers the opportunity to study the molecular switches that may regulate or be regulated by the mechanical changes in 3D co-cultures. Only studies of perturbations induced over time will be able to distinguish cause and effect for key molecular effectors such as HGF (hepatocyte growth factor)-signaling [38]. In such studies, the variation of matrix stiffness can be achieved by varying the collagen I concentration in the Matrigel:collagen I mixture [34], [40]. Our current study in a well-characterized, progressive 3D co-culture series, establishes OCT as a convenient platform for such future studies.

Future studies would also benefit from merging longitudinal evaluation of morphology with studies of RNA and protein expression from whole genome microarrays performed using a bioinformatics approach [9]. Previous work by Kenny, *et al,* correlated four distinct morphologies of MEC colonies (round, mass, grape-like, and stellate) with gene expression [13], although no data is yet available to show time- or co-cultured fibroblast-dependent morphological changes. Morphological characteristics are likely parallel to molecular phenotypic changes, and an imaging-based biomarker of shifts in molecular phenotype could allow advances in our understanding of the physical and mechanical regulation of molecular signaling. For example, previous xenograft studies have shown that MCF10DCIS.com cells are more invasive than MCF10A cells, and are enriched for expression of lymphangiogenesis markers [41]. These xenografts highlight that the breast cancer microenvironment is comprised of many cell types, and while the fibroblast is a highly abundant stromal cell type, there are many other possible contributors and mediators of the complex paracrine communication in breast tissue. However, the simplified 3D model of MEC and fibroblasts mirrors xenografts in its ability to track acini growth and asphericity, and therefore may be an *in vitro* approach to studying invasive potential.

We also note that the association between premalignant cells and high asphericity observed in this study may be related to previous studies establishing a connection between tissue structural complexity (in a mathematical sense, such as fractal dimension) and various cancers [42]–[44]. While asphericity is not a measure of complexity *per se*, acini with a high fractal dimension would be expected to have a high asphericity. Tying the efforts reported here in engineered tissues with OCT imaging and morphological analysis of real breast cancer tissues [22], [23], [26], [27] may lead to new mechanistic insight, and may also translate to clinical OCT imaging efforts, such as those in surgical guidance during breast cancer surgery [21].

## Supporting Information

Figure S1 **Schematic diagram of the OCT system.** The ultrahigh resolution SD-OCT system is comprised of a Ti:Sapphire laser, a Michelson interferometer, and a high speed spectrometer (details in text). FS: fiber to free-space coupler, SF: free-space to fiber coupler.
(TIF)

Figure S2 **Representative OCT x-z images of 3D human mammary tissue cultures at week 2.** As indicated, the seed concentration of MEC is increasing from top to bottom, and the seed concentration of RMF is increasing from left to right.
(TIF)

Figure S3 **Representative OCT x-z images of 3D human mammary tissue cultures at week 4.** As indicated, the seed concentration of MEC is increasing from top to bottom, and the seed concentration of RMF is increasing from left to right.
(TIF)

Figure S4 **Acini size analysis.** Histogram of average acini sizes (in µm^2^) in each gel formed by the normal and pre-malignant MECs, based on the OCT images acquired weekly for 4 weeks. Error bars indicate the standard error of the measured values.
(TIF)

Figure S5 **Number of MCF10A and MCF10DCIS.com cells in acini with increasing fibroblasts.** Lack of proliferation between week 2 and week 3 is evident from the decrease in number of MEC per acinus.
(TIF)

Table S1 **Number of acini in 3D cultures at week 4.** Acini count in an approximate gel volume of 4.5 mm^3^ at week 4 for monocultures of MCF10A and MCF10DCIS.com, and co-cultures of MCF10A:RMF and MCF10DCIS.com:RMF.
(DOC)

File S1 **Supplementary.**
(DOC)
