# Supplementary figures and images for: Longitudinal Study of Mammary Epithelial and Fibroblast Co-Cultures Using Optical Coherence Tomography Reveals Morphological Hallmarks of Pre-Malignancy
Source: PLoS One. 2012 Nov 12;7(11):e49148. doi: 10.1371/journal.pone.0049148 (PMC3495770; doi:10.1371/journal.pone.0049148)

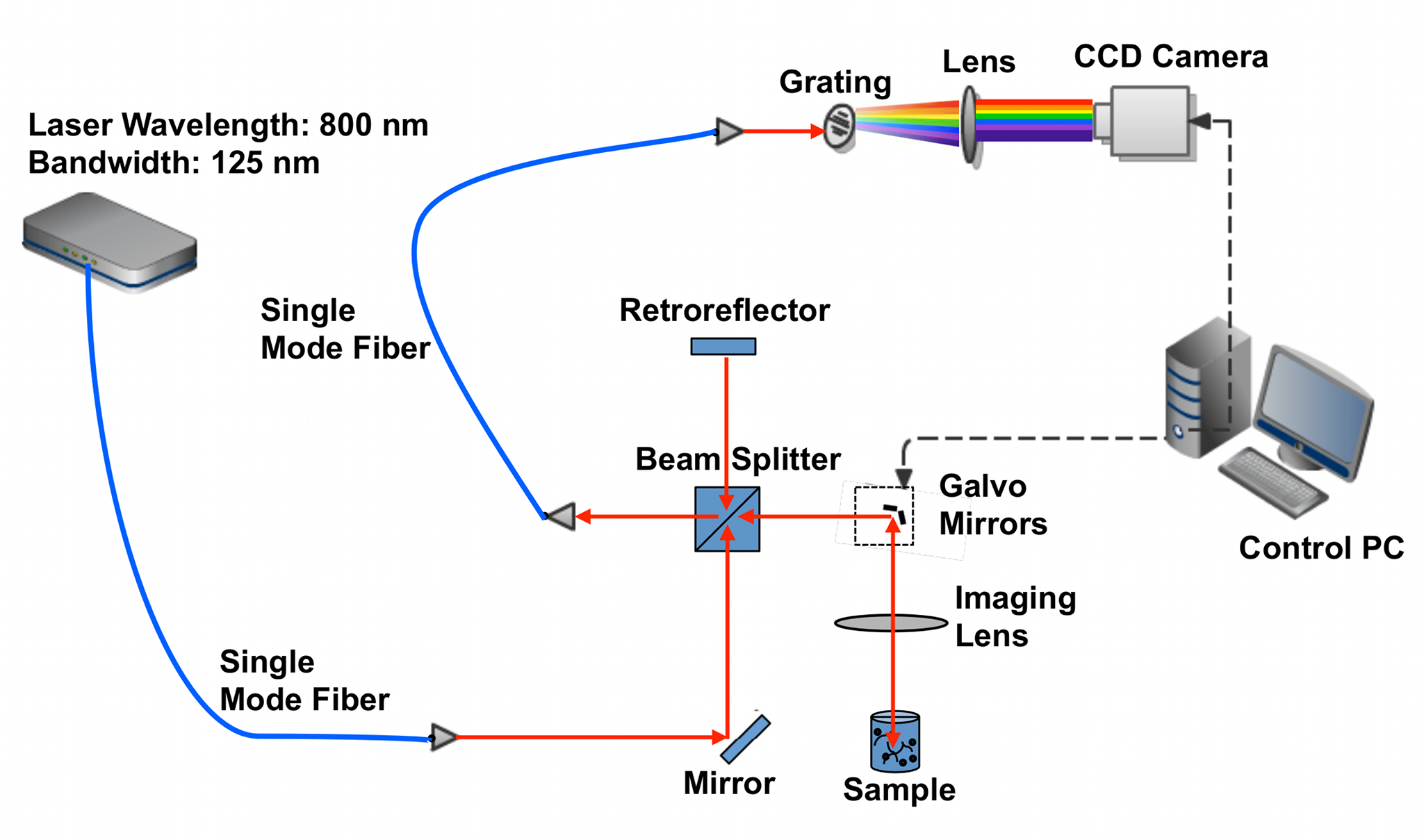

Supplement: Figure S1 — Schematic diagram of the OCT system. The ultrahigh resolution SD-OCT system is comprised of a Ti:Sapphire laser, a Michelson interferometer, and a high speed spectrometer (details in text). FS: fiber to free-space coupler, SF: free-space to fiber coupler. (TIF) [file pone.0049148.s001.tif]

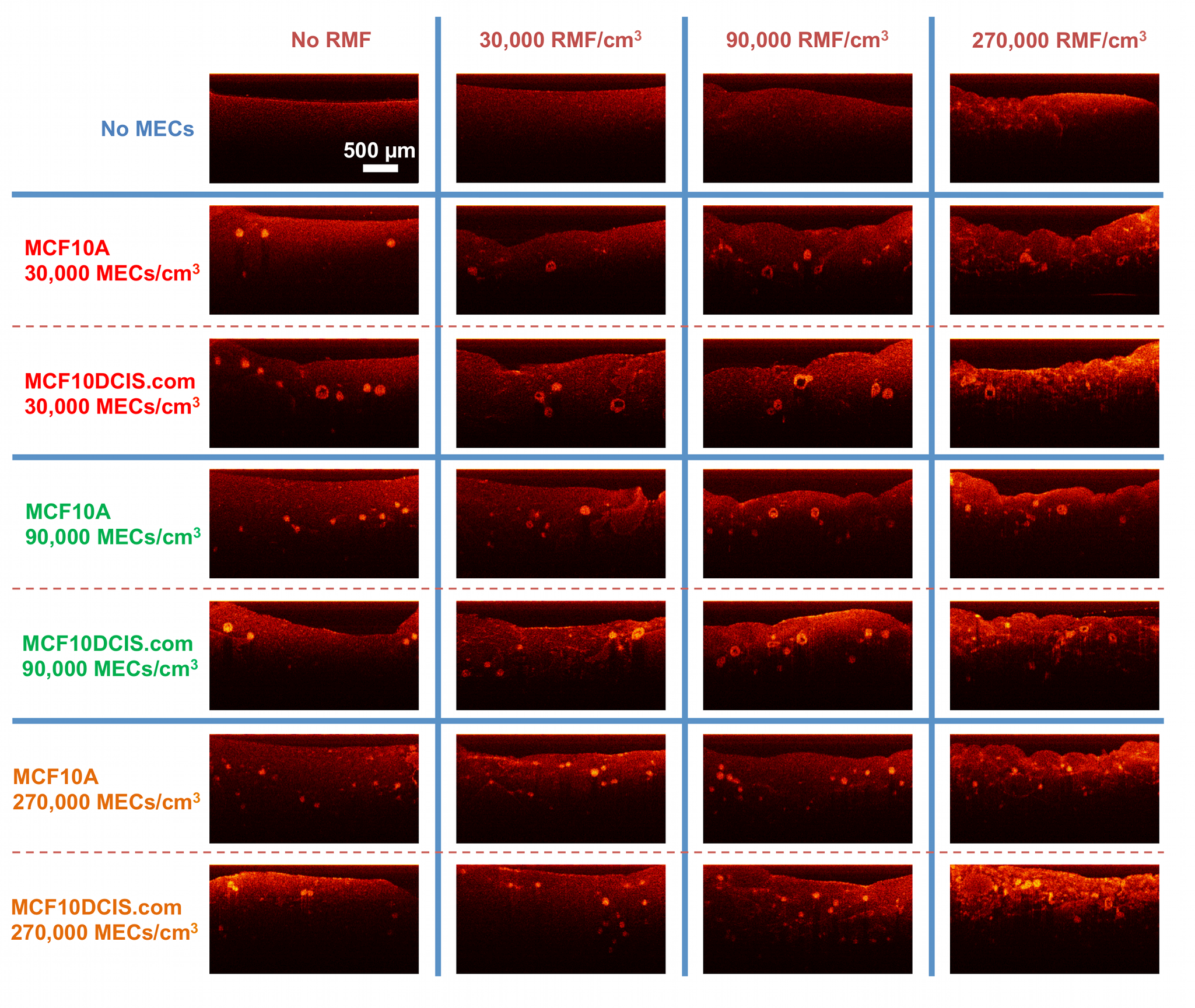

Supplement: Figure S2 — Representative OCT x-z images of 3D human mammary tissue cultures at week 2. As indicated, the seed concentration of MEC is increasing from top to bottom, and the seed concentration of RMF is increasing from left to right. (TIF) [file pone.0049148.s002.tif]

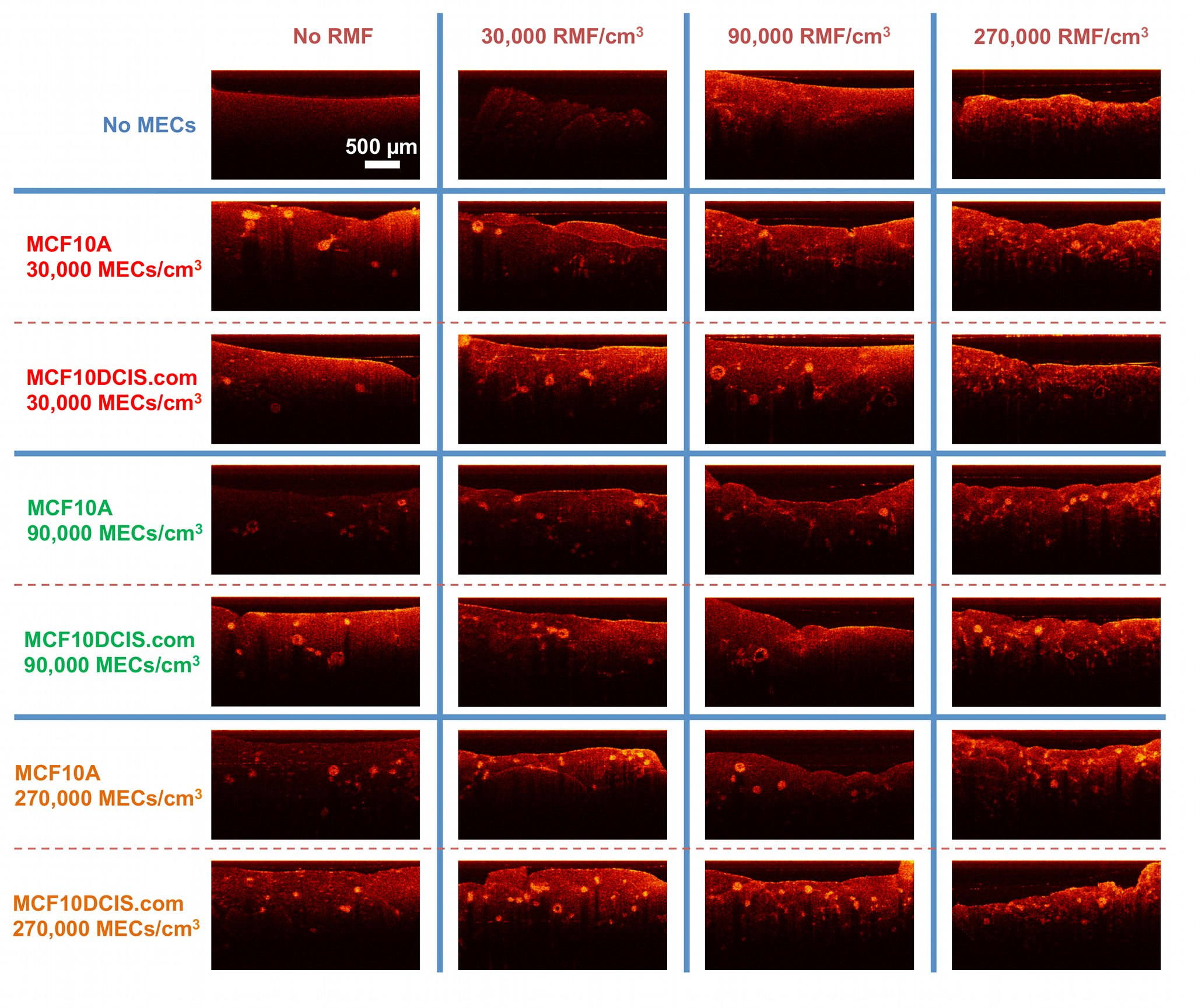

Supplement: Figure S3 — Representative OCT x-z images of 3D human mammary tissue cultures at week 4. As indicated, the seed concentration of MEC is increasing from top to bottom, and the seed concentration of RMF is increasing from left to right. (TIF) [file pone.0049148.s003.tif]

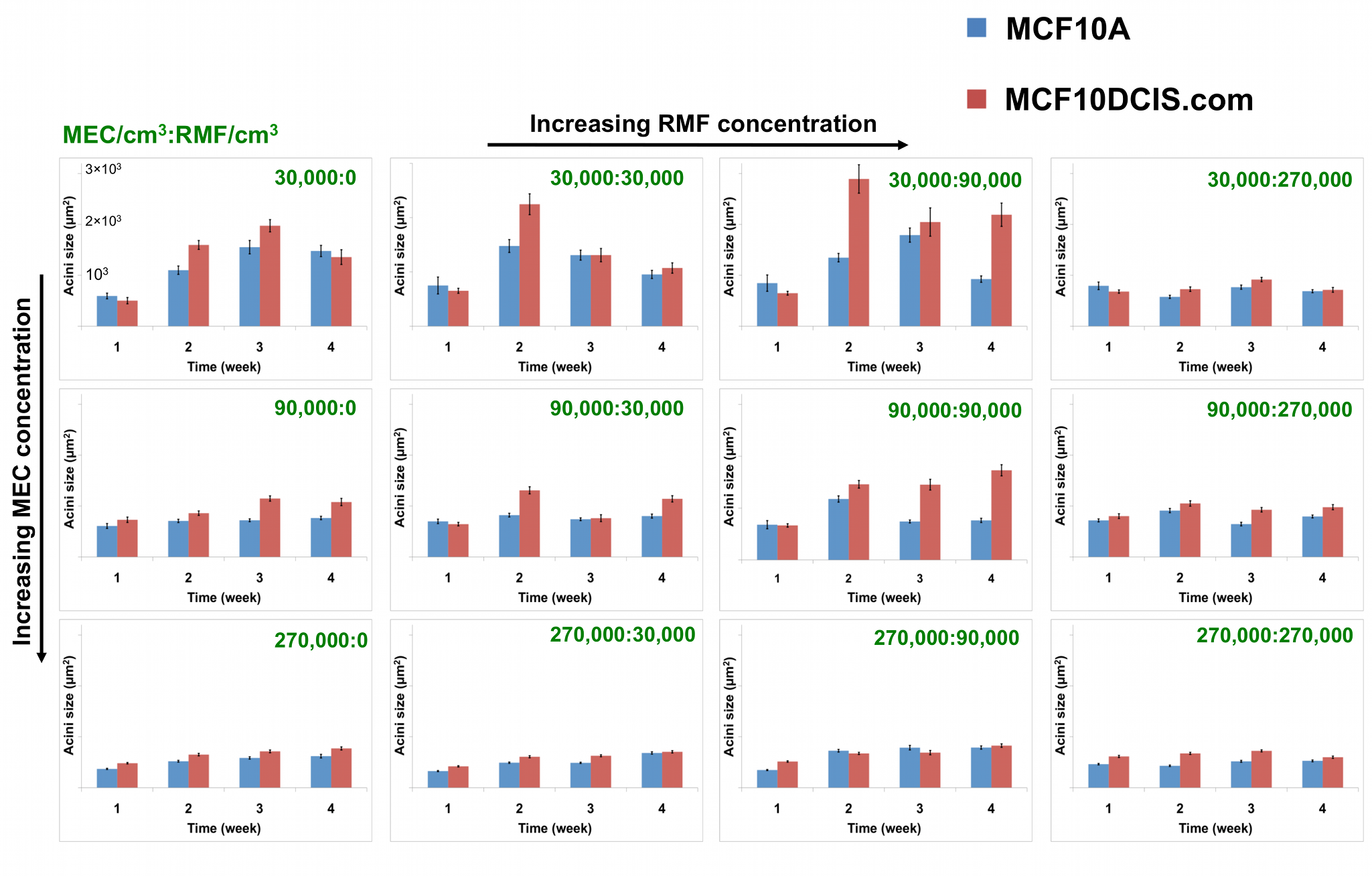

Supplement: Figure S4 — Acini size analysis. Histogram of average acini sizes (in µm2) in each gel formed by the normal and pre-malignant MECs, based on the OCT images acquired weekly for 4 weeks. Error bars indicate the standard error of the measured values. (TIF) [file pone.0049148.s004.tif]

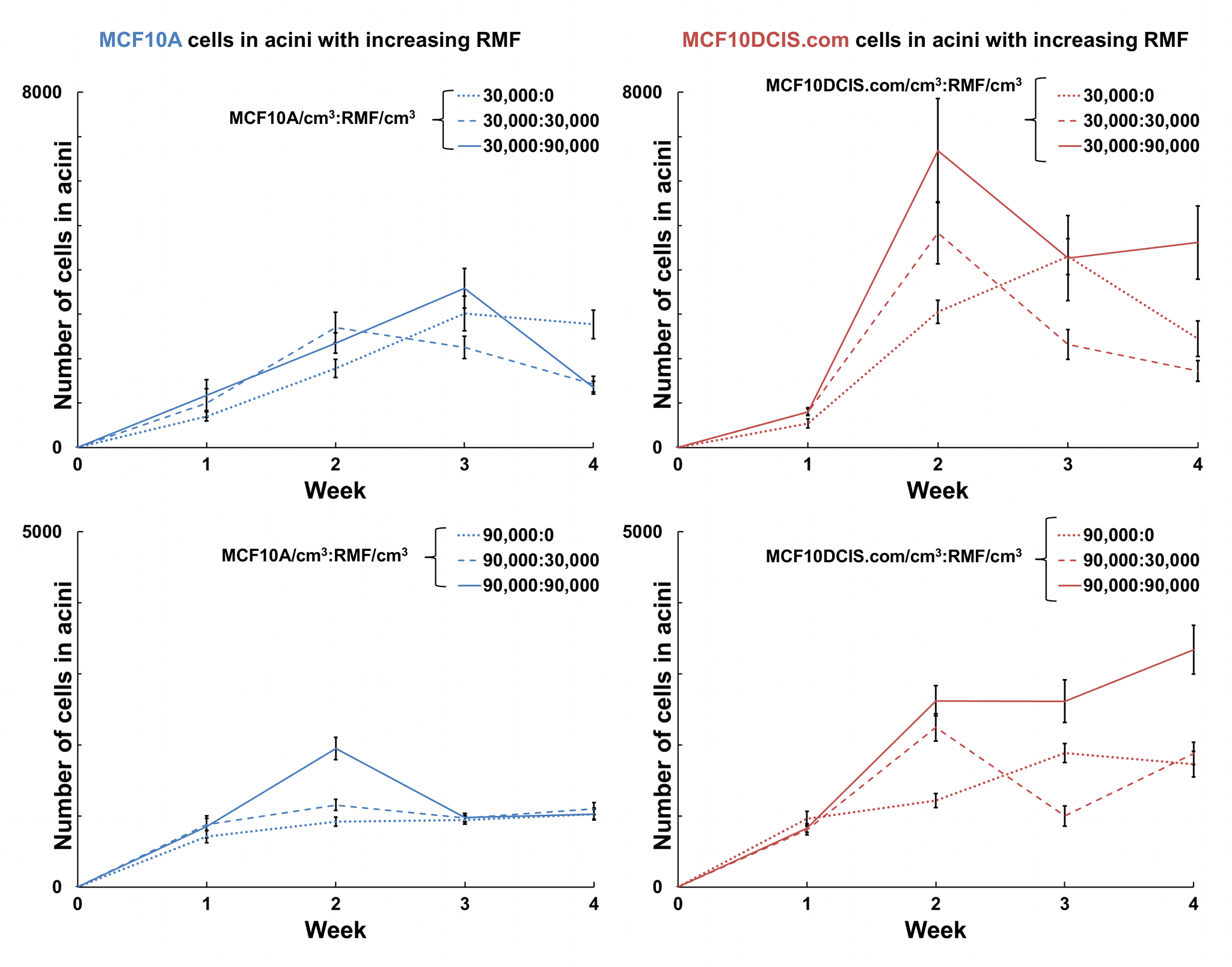

Supplement: Figure S5 — Number of MCF10A and MCF10DCIS.com cells in acini with increasing fibroblasts. Lack of proliferation between week 2 and week 3 is evident from the decrease in number of MEC per acinus. (TIF) [file pone.0049148.s005.tif]
