# Supplementary material for: Longitudinal Study of Mammary Epithelial and Fibroblast Co-Cultures Using Optical Coherence Tomography Reveals Morphological Hallmarks of Pre-Malignancy
Source: PLoS One. 2012 Nov 12;7(11):e49148. doi: 10.1371/journal.pone.0049148 (PMC3495770; doi:10.1371/journal.pone.0049148)
Supplement: Table S1 — Number of acini in 3D cultures at week 4. Acini count in an approximate gel volume of 4.5 mm3 at week 4 for monocultures of MCF10A and MCF10DCIS.com, and co-cultures of MCF10A:RMF and MCF10DCIS.com:RMF. (DOC) [file pone.0049148.s006.doc]

## Supplementary Table

Table S1: **Number of acini in 3D cultures at week 4**

|  | **No RMF** | **30,000 RMF/cm^3^** | **90,000 RMF/cm^3^** | **270,000 RMF/cm^3^** |
| --- | --- | --- | --- | --- |
| **30,000 MCF10A/cm^3^** | 45 | 52 | 66 | 184 |
| **30,000 MCF10DCIS.com/cm^3^** | 20 | 39 | 46 | 123 |
| **90,000 MCF10A/cm^3^** | 119 | 96 | 66 | 199 |
| **90,000 MCF10DCIS.com/cm^3^** | 75 | 61 | 56 | 187 |
| **270,000 MCF10A/cm^3^** | 183 | 174 | 94 | 231 |
| **270,000 MCF10DCIS.com/cm^3^** | 152 | 279 | 139 | 197 |

Acini count in an approximate gel volume of 4.5 mm^3^ at week 4 for monocultures of MCF10A and MCF10DCIS.com, and co-cultures of MCF10A:RMF and MCF10DCIS.com:RMF.
