# Supplementary material for: Longitudinal Study of Mammary Epithelial and Fibroblast Co-Cultures Using Optical Coherence Tomography Reveals Morphological Hallmarks of Pre-Malignancy
Source: PLoS One. 2012 Nov 12;7(11):e49148. doi: 10.1371/journal.pone.0049148 (PMC3495770; doi:10.1371/journal.pone.0049148)
Supplement: File S1 — Supplementary. (DOC) [file pone.0049148.s007.doc]

# Supplementary

## Longitudinal Study of Mammary Epithelial & Fibroblast Co-Cultures using Optical Coherence Tomography Reveals Morphological Hallmarks of Pre-Malignancy

Raghav K. Chhetri^1^, Zachary F. Phillips^2^, Melissa A. Troester^3^, Amy L. Oldenburg^1,4*^

^1^Department of Physics and Astronomy, University of North Carolina at Chapel Hill, Chapel Hill, NC, USA

^2^Curriculum in Applied Sciences and Engineering, University of North Carolina at Chapel Hill, Chapel Hill, NC, USA

^3^Lineberger Comprehensive Cancer Center/Department of Epidemiology, University of North Carolina at Chapel Hill, Chapel Hill, NC, USA

^4^Biomedical Research Imaging Center, University of North Carolina at Chapel Hill, Chapel Hill, NC, USA

* E-mail: [aold@physics.unc.edu](mailto:aold@physics.unc.edu)

## OCT System Hardware

Imaging of the 3D cultures was performed using a custom, ultrahigh-resolution, spectral-domain optical coherence tomography (SD-OCT) system which employed a low-coherence light source consisting of a Ti:Sapphire laser (Griffin, KMLabs, Inc.) with a central wavelength of 800nm and a bandwidth of 125 nm. The axial (depth, *z*) resolution of the imaging system owing to the wavelength and the bandwidth of the light source is 3 μm in air. Single-mode fibers were employed to simplify alignment between the components of the OCT system (laser, interferometer, and spectrometer). The light source was directed into a free-space Michelson interferometer comprised of a stationary reference arm and a sample (imaging) arm. In the sample arm, 3D cultures were illuminated by a 10mW beam focused by a 30 mm focal length achromatic lens, which provided a resolution of 12 μm (air) in the transverse (*x* and *y*) directions. Transverse raster-scanning over the sample was achieved using galvanometer-controlled mirrors. The backscattered light from the sample was collected by the same illuminating lens and interfered with the reference beam at the output of the interferometer. The interfered beam was directed into a custom-bulit spectrometer, which consists of a diffraction grating (600 lines/mm), imaging lens (focal length 200 mm), and a 25 kHz CCD linescan camera (Piranha, Dalsa Inc.). The spectral interferograms recorded by the camera are related to the depth-dependent backscattering potential of the sample via Fourier transformation, as understood for SD-OCT systems [1]. Data processing of the spectral interferogram was performed according to our previously published methods [2] to produce an OCT image with an imaging depth of 2 mm in air.

## OCT **Imaging Results**

Representative OCT images of monocultures and co-cultures at each culture condition are shown in Figures S2 and S3 at weeks 2 and 4, respectively. Gels that had higher seed concentrations of MEC and/or RMF were observed to have higher acini counts (Table S1) but smaller overall acini sizes (Figure S4). This suggests that the acini growth is stunted by higher seed concentrations due to resource scarcity or cellular crowding.

To estimate the number of MEC per acinus, the total volume occupied by cells was approximated from the measured acini and lumen areas, *A_acini_* and *A_lumen_*, and compared to the volume of each MEC, *V_cell_*, leading to the following relation:

$$\text{Number} \text{of} \text{MEC} \text{per} \text{acinus} =0.74\times\left[\frac{\left(\frac{4}{3\sqrt{\pi}}\right)\times\left(A_{acini}^{3/2}-A_{lumen}^{3/2}\right)}{V_{cell}}\right]$$

where 0.74 is the volume packing fraction for spheres, and the MEC were approximated to be spherical. The individual MEC were found to have an average radius of 4.37 μm from fluorescent images of acini in the co-cultures, resulting in an estimation of *V_cell_* as 350 μm^3^. Thus the number of MEC per acinus was computed by dividing the total volume occupied by MEC by the volume of each MEC. In estimating the number of MEC per acini, we assume that acini are predominantly comprised of MECs.

Figure S5 shows the average number of cells in acini over 4 weeks. Between week 2 and week 3, proliferation of the cells in the co-cultures was no longer in the log-phase of growth, as evidenced by a plateau in the number of MEC per acinus. Similarly, co-cultures with higher seeded cell concentration (MEC concentration > 90,000/cm^3^) also remained in the log-phase for only a short time (Figure S5) and the acini count shows numerous smaller acini interspersed in the Collagen I:Matrigel matrix (Table S1). Thus, monocultures seeded with 30,000 MEC/cm^3^, co-cultures seeded with 30,000 MEC/cm^3^:30,000 RMF/cm^3^, and 30,000 MEC/cm^3^:90,000 RMF/cm^3^ were selected for comparison over the first two weeks only.

## References

1. Fercher AF, Hitzenberger CK, Kamp G, El-Zaiat SY (1995) Measurement of intraocular distances by backscattering spectral interferometry. Optics Communications 117: 43–48.

2. Oldenburg AL, Gallippi CM, Tsui F, Nichols TC, Beicker KN, et al. (2010) Magnetic and contrast properties of labeled platelets for magnetomotive optical coherence tomography. Biophysical Journal 99: 2374–2383.
